# Acute Scrotum Masquerading as Testicular Torsion: Spontaneous Intratesticular Hematoma in a Patient With Congenital Afibrinogenemia

**DOI:** 10.7759/cureus.115633

**Published:** 2026-09-02

**Authors:** Hussein Zein Alabidin Almasri, Asaad Skoury

**Affiliations:** 1 Department of Emergency Medicine, American University of Beirut Medical Center, Beirut, LBN

**Keywords:** acute scrotum, congenital afibrinogenemia, cremasteric reflex, fibrinogen concentrate, intratesticular hematoma, rare bleeding disorder, testicular torsion, twist score

## Abstract

Congenital afibrinogenemia is an exceedingly rare autosomal recessive bleeding disorder characterized by the complete absence of circulating fibrinogen, predisposing affected individuals to spontaneous and trauma-related hemorrhage at virtually any anatomical site. Acute scrotal pain, by contrast, is a common urological emergency in which testicular torsion must be excluded promptly, as diagnostic delay risks irreversible testicular loss. We describe a 31-year-old man with known congenital afibrinogenemia, maintained on periodic fibrinogen concentrate replacement, who presented with acute right testicular and inguinal pain out of proportion to initial findings, accompanied by nausea. Examination revealed a tender, swollen, firm, high-riding right testis with an absent cremasteric reflex, a constellation corresponding to the maximum possible Testicular Workup for Ischemia and Suspected Torsion (TWIST) score of seven points and strongly suggestive of torsion. Manual detorsion was attempted, with partial symptomatic improvement. Scrotal Doppler ultrasonography, performed after an attempted manual detorsion with partial symptomatic improvement, demonstrated preserved, symmetric flow with no evidence of torsion at the time of imaging, alongside avascular, hypoechoic, geographic intratesticular regions most consistent with intratesticular hematoma in the context of the patient's coagulopathy. Multidisciplinary management by emergency medicine, urology, and hematology favored conservative treatment with intravenous fibrinogen concentrate and analgesia over surgical exploration, given both the sonographic findings of preserved perfusion and no evidence of torsion and the prohibitive hemorrhagic risk that surgery would pose in a patient with a complete absence of fibrinogen. The patient was discharged with a plan for follow-up imaging and multidisciplinary outpatient review. This case illustrates that spontaneous intratesticular hemorrhage can closely mimic testicular torsion on history and examination, even fulfilling maximal clinical risk-scoring criteria, and underscores the pivotal role of prompt Doppler ultrasonography in guiding safe, conservative management in patients for whom surgery carries extreme bleeding risk. Awareness of this rare mimicker is essential for clinicians managing acute scrotal pain in patients with severe inherited coagulopathies.

## Introduction

Acute scrotal pain is a common presentation to the emergency department (ED) and represents one of the few true urological emergencies. Testicular torsion, though responsible for only a minority of acute scrotum presentations, must be excluded rapidly in every case, as testicular salvage rates fall sharply once ischemia extends beyond several hours [1]. Clinical assessment, including testicular position, consistency, tenderness, and the cremasteric reflex, has traditionally guided initial risk stratification, and structured tools such as the Testicular Workup for Ischemia and Suspected Torsion (TWIST) score have been developed and validated, in children and more recently in adults, to standardize this assessment [2]. Nevertheless, clinical findings alone cannot reliably exclude torsion, and color Doppler ultrasonography remains the imaging modality of choice, combining high sensitivity and specificity for torsion with the ability to identify alternative intrascrotal pathology [3].

Congenital afibrinogenemia is an exceedingly rare autosomal recessive disorder, estimated to affect fewer than one in a million individuals, resulting from mutations in the fibrinogen genes FGA, FGB, or FGG and leading to a complete or near-complete absence of circulating fibrinogen [4]. Its clinical course is markedly heterogeneous, ranging from mucocutaneous and joint bleeding to life-threatening intracranial, gastrointestinal, or solid-organ hemorrhage, and it paradoxically also carries an increased thrombotic risk [4]. Genitourinary and testicular bleeding have been described only sporadically in this population [5].

Because spontaneous or minor-trauma intratesticular hemorrhage can produce a clinical picture that closely resembles testicular torsion, patients with severe inherited coagulopathies pose a particular diagnostic and management challenge: an erroneous decision to proceed directly to surgical exploration could expose a patient with negligible clotting capacity to catastrophic intraoperative or postoperative hemorrhage. We report the case of a young man with congenital afibrinogenemia who presented with an acute scrotum clinically indistinguishable from testicular torsion, in which Doppler ultrasonography proved essential in identifying findings most consistent with an intratesticular hematoma and averting unnecessary surgery.

## Case presentation

A 31-year-old man with a body mass index of 22 kg/m² and a known history of congenital afibrinogenemia, maintained on periodic intravenous fibrinogen concentrate replacement (most recent infusion 20 days prior to presentation), presented to the ED with acute right testicular pain. He was also a current smoker. The pain had begun approximately one to two days earlier as a mild ache in the right testicular and inguinal region but had become markedly more severe and out of proportion to findings over the preceding two to three hours. The pain was sharp, radiated to the right inguinal and flank areas, and was associated with nausea and abdominal discomfort. He denied fever, chills, urinary symptoms, recent trauma, and recent sexual intercourse.

On further questioning, the patient recalled a similar episode several years earlier, when he developed a testicular hematoma after being struck by a friend with moderate force; this had been managed conservatively. He also described intermittent minor bleeding from the urethral meatus following intercourse, previously attributed to minor coital trauma, for which he and his partner had adopted a cautious approach to intercourse.

On examination, the patient was in severe distress, reportedly screaming in pain. Vital signs were notable for a blood pressure of 153/90 mmHg, heart rate of 80 beats/min, respiratory rate of 20/min, temperature of 36.8°C, and oxygen saturation of 100% on room air. Genitourinary examination revealed a normal penis. The right testis was tender, swollen, firm, and high-riding, with an absent cremasteric reflex; the left testis was unremarkable, though later noted to be borderline small in comparison. This constellation, testicular swelling, a firm testis, an absent cremasteric reflex, a high-riding position, and nausea, corresponds to the maximum possible score of seven points on the TWIST scale [2].

Given the clinical impression of acute testicular torsion, the ED attending performed one attempt at manual detorsion, which produced partial symptomatic improvement. Initial laboratory results (Table 1) were largely unremarkable, with a mildly elevated absolute neutrophil count as the only abnormality; urinalysis showed no evidence of infection or hematuria, with an alkaline pH and occasional amorphous phosphate crystals and hyaline casts felt to be nonspecific. A fibrinogen level was not obtained at this presentation prior to treatment, as the patient's diagnosis and severity of deficiency were already well-established; empiric replacement was therefore initiated based on his known history without repeat baseline testing.

**Table 1:** Initial laboratory investigations on presentation (H): value flagged by the laboratory as abnormally high; WBC: white blood cell count; RBC: red blood cell count; HGB: hemoglobin; HCT: hematocrit; ANC: absolute neutrophil count

Parameter	Reference range	Result
Adjusted WBC	4,000-11,000/cu.mm	9,900
RBC	4.50-6.50 mil/cu.mm	6.09
HGB	13.0-18.0 g/dL	16.7
HCT	40-54%	50
Platelets	150,000-400,000/cu.mm	241,000
ANC	1,600-7,200/cu.mm	7,227 (H)

Scrotal Doppler ultrasonography was subsequently performed (Figure 1). It demonstrated avascular, hypoechoic, geographic regions within the right testicle, most consistent with intratesticular hematoma in the context of the patient's known afibrinogenemia; the tunica albuginea was preserved, and the testicle appeared engorged but without features suggestive of a solid neoplasm. Both testes demonstrated normal, symmetric Doppler flow, and the left testis was otherwise sonographically normal. An incidental 8-mm left epididymal cyst and small bilateral hydroceles were also noted. The formal impression was that there was no sonographic evidence of testicular torsion at the time of examination, with findings instead favoring intratesticular hematoma.

**Figure 1:**
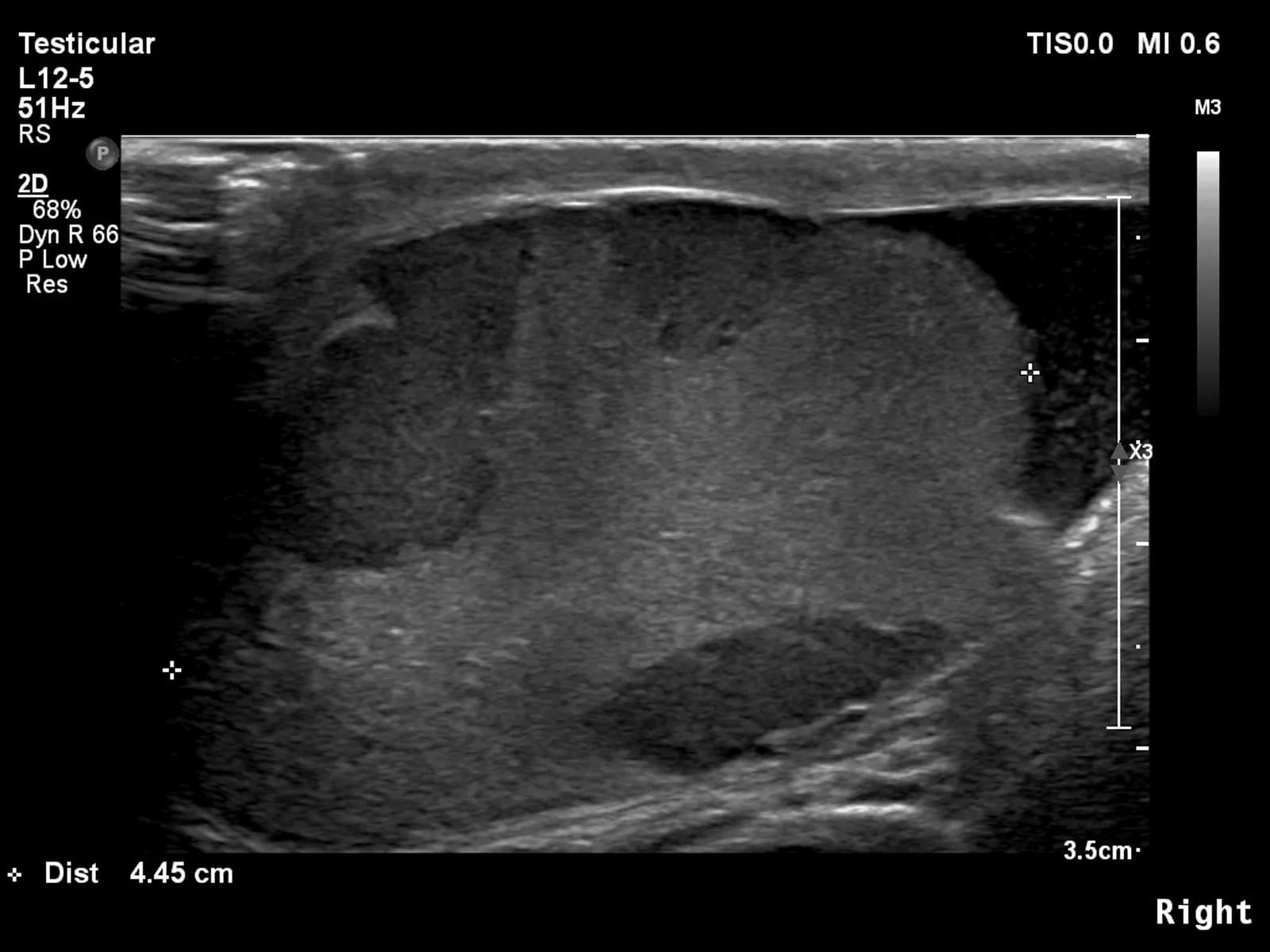
Scrotal ultrasound of the right testis demonstrating an avascular, hypoechoic, geographic region within the testicular parenchyma, consistent with intratesticular hematoma, with preserved tunica albuginea

Urology and hematology were both consulted. The urology team assessed that the patient's ongoing pain was attributable to the intracapsular (intratesticular) bleed itself and recommended conservative management without surgical intervention. On subsequent reassessment, the patient's pain had partially improved with analgesia but had not fully resolved. Inpatient admission for observation was initially planned; this was ultimately not pursued because of a lack of insurance coverage, though the multidisciplinary team agreed that outpatient management was a medically reasonable alternative given the sonographic findings of preserved perfusion and no evidence of torsion, the fibrinogen concentrate administered, and the close follow-up arranged with urology and hematology. The patient elected to be discharged on this basis. He received a single dose of intravenous fibrinogen concentrate (4 g) in the ED, together with analgesia, and was discharged with a plan for outpatient follow-up scrotal ultrasonography in two weeks and clinical follow-up with both the urology and hematology teams.

## Discussion

This case illustrates two related challenges: the clinical overlap between testicular torsion and its mimickers, and the amplified stakes of diagnostic error in a patient with a severe inherited coagulopathy. Our patient's presentation-acute, severe, out-of-proportion pain; a swollen, firm, high-riding testis; an absent cremasteric reflex; and associated nausea-reproduced the complete set of criteria used in the TWIST score, yielding the maximum possible score of seven points. In the adult validation cohort for this tool, scores of 6 or 7 carried a positive predictive value of 100% for surgically confirmed torsion [2].

Despite this, ultrasonography demonstrated preserved, symmetric perfusion and no evidence of torsion at the time of imaging in our patient. This discrepancy is a useful reminder that structured clinical scores, however well-validated, are derived from cohorts that have explicitly excluded patients with prior scrotal pathology [2] and, in practice, are unlikely to have enrolled patients with known bleeding disorders; their performance therefore cannot be assumed to generalize to this population.

One alternative explanation deserves explicit discussion: because Doppler ultrasonography was performed after an attempted manual detorsion, the preserved, symmetric flow observed establishes the absence of torsion at the time of imaging but cannot, on its own, exclude a preceding torsion-detorsion event or intermittent torsion. Two features of the clinical course argue against this possibility. First, the improvement following detorsion was partial rather than complete, whereas successful correction of true torsion typically produces prompt and substantial symptomatic relief. Second, on reassessment the patient continued to have significant pain without further improvement, a trajectory more consistent with an ongoing process such as an expanding hematoma than with a mechanically corrected torsion. Nonetheless, in the absence of surgical exploration or imaging prior to the detorsion attempt, a preceding torsion-detorsion event cannot be entirely excluded on the available evidence, and we have revised our language throughout the manuscript accordingly. Similarly, while an absent cremasteric reflex is a sensitive finding in torsion, it lacks specificity and can be present in a range of other painful scrotal conditions [1]; in our patient, it more likely reflected pain and local swelling than cord ischemia.

Color Doppler ultrasonography remains the imaging investigation of choice for the acute scrotum, combining high diagnostic accuracy for torsion with the ability to characterize alternative pathology [3]. In our patient, it demonstrated avascular, hypoechoic, geographic intratesticular regions with preserved, symmetric perfusion, a pattern well-recognized as the sonographic signature of intratesticular hematoma, which typically appears as an avascular, hypoechoic, ovoid or crescent-shaped lesion, often with a thin peripheral rim of hyperemia, and can be difficult to distinguish from segmental infarction or an underlying neoplasm without further characterization [6]. Because this entity is uncommon and easily mistaken for other focal intratesticular pathology, it is thought to be underrecognized, and misdiagnosis has historically led to unnecessary orchiectomy [6].

Congenital afibrinogenemia's bleeding phenotype is well-described in general terms [4,7], but genitourinary and testicular bleeding are reported only sporadically. Testicular hematoma has previously been documented as part of a multiorgan hemorrhagic phenotype in a patient with this disease [5], spontaneous hemorrhage into other solid organs, such as the liver, has likewise been reported [8], and afibrinogenemia-related bleeding has been the subject of several previous case reports, including within this journal [9]. Our patient's own history of a prior traumatic testicular hematoma and recurrent minor postcoital urethral bleeding suggests an individual predisposition toward bleeding at the genital and lower urinary tract. This observation may be of practical relevance when counseling similar patients about activity-related trauma. His most recent prophylactic fibrinogen infusion had been administered 20 days before presentation, and it is plausible that a relative decline in circulating fibrinogen between scheduled doses contributed to bleeding at the time of this otherwise unprovoked episode; however, as a contemporaneous fibrinogen level was not obtained at this presentation, this relationship cannot be assessed directly and remains a plausible but unconfirmed hypothesis.

The distinction between torsion and hematoma was not merely academic in this patient. Surgical exploration is the standard of care whenever torsion cannot be confidently excluded, but subjecting a patient with a complete absence of circulating fibrinogen to surgery would carry a substantially elevated risk of uncontrolled intraoperative and postoperative hemorrhage. Once torsion was excluded sonographically, the multidisciplinary team was able to pursue conservative management, targeted fibrinogen concentrate replacement and analgesia, in line with established dosing principles for congenital fibrinogen deficiency, which recommend replacement sufficient to achieve and maintain fibrinogen activity above hemostatic thresholds for non-surgical bleeding [10]. This management strategy avoided exposing the patient to an operation whose diagnostic yield was limited once imaging had already demonstrated preserved perfusion and no evidence of torsion at the time of assessment, the diagnosis for which surgery is truly time-critical.

This report carries the inherent limitations of a single case. Because the planned follow-up ultrasound was not available at the time of writing, interval resolution of the lesion was not documented, and the diagnosis of hematoma therefore rests on the initial clinical and sonographic findings rather than longitudinal radiographic confirmation. Additionally, because Doppler ultrasonography was performed only after an attempted manual detorsion, a preceding torsion-detorsion event cannot be entirely excluded; the partial, rather than complete, symptomatic response to detorsion argues against this, though it cannot be ruled out definitively on the available data. The timing of the patient's discharge, ahead of the initially planned admission, was influenced by a lack of insurance coverage, although the multidisciplinary team judged outpatient management to be a reasonable alternative on clinical grounds; outcome data beyond the immediate ED course were not available at the time of writing. Nonetheless, this case adds to a very small body of literature documenting testicular or scrotal hemorrhage as a manifestation of congenital afibrinogenemia and reinforces a practical point for clinicians: in patients with known severe coagulopathy who present with an acute scrotum, however convincing the examination for torsion, prompt Doppler ultrasonography should be pursued before proceeding to surgery whenever the clinical situation allows, since the diagnostic and therapeutic stakes of getting this distinction right are unusually high in this population.

## Conclusions

Spontaneous or minor-trauma intratesticular hemorrhage can present with a clinical picture indistinguishable from testicular torsion, even fulfilling the maximum criteria of validated clinical risk scores. In patients with severe inherited coagulopathies such as congenital afibrinogenemia, this distinction carries particular weight, since surgical exploration, the default management for suspected torsion, poses a substantially elevated bleeding risk. Prompt color Doppler ultrasonography was essential in this case to demonstrate preserved perfusion and no evidence of torsion at the time of imaging, identify findings most consistent with an intratesticular hematoma, and support safe, conservative management with fibrinogen replacement. Clinicians managing acute scrotal pain in patients with known bleeding disorders should maintain a high index of suspicion for this rare but clinically important mimicker.
